# Case report: Chronic disseminated intravascular coagulopathy with concurrent paraneoplastic secondary hyperfibrinolysis in a dog with metastatic nasal adenocarcinoma

**DOI:** 10.3389/fvets.2024.1375507

**Published:** 2024-05-17

**Authors:** Kyle L. Granger, Trish Paulos, Mary-Keara Boss, Liz Guieu, Sarah Shropshire

**Affiliations:** Department of Clinical Sciences, College of Veterinary Medicine and Biomedical Sciences, Colorado State University, Fort Collins, CO, United States

**Keywords:** paraneoplastic, DIC, secondary hyperfibrinolysis, adenocarcinoma, dog

## Abstract

In human medicine, hemostatic disorders such as thrombocytopenia, hyperfibrinolysis, and disseminated intravascular coagulopathy (DIC) have been associated with many cancers. Acute hemorrhage secondary to hyperfibrinolysis has been predominantly reported with prostatic adenocarcinoma in human patients. To the author’s knowledge, severe bleeding due to paraneoplastic hyperfibrinolysis has not yet been reported in veterinary medicine. The case involves an 8-year-old neutered male Border Collie who was evaluated for progressive and recurrent epistaxis, having a history of 1 year of treatment for metastatic nasal adenocarcinoma. A progressive and severe coagulopathy thought to be related to the known cancer was diagnosed. Advanced coagulation testing was consistent with a chronic DIC and secondary hyperfibrinolysis. Throughout 1 week of hospitalization, the dog was treated with multiple blood products, vitamin K_1_, and anti-fibrinolytic medications. While the dog was initially discharged home, the dog re-presented the following day and was humanely euthanized due to a perceived poor quality of life. Post-mortem analysis revealed a histopathologic diagnosis of disseminated adenocarcinoma. In dogs with disseminated nasal adenocarcinoma that are experiencing severe bleeding, paraneoplastic secondary hyperfibrinolysis should be considered as a differential. Knowing this association could help guide treatment recommendations for optimal patient management.

## Introduction

Though not completely understood, the interplay between neoplastic processes and the coagulation system is characterized by an imbalance between procoagulant and anticoagulant factors (1). Currently, it is theorized that the hemostatic system gets activated by procoagulant factors such as tissue factor that is produced by cancer cells. This activation results in local production of thrombin leading to fibrin deposition and platelet recruitment and ultimately resulting in thrombosis and consumption of coagulation factors (2). Paraneoplastic hyperfibrinolysis is thought to occur in two ways; the tumor cells either (A) produce profibrinolytic proteins, such as urokinase-type plasminogen activator (uPA) and tissue-type plasminogen activator (tPA) or (B) tumor cell membranes carry the specific uPA receptor which can trigger the assembly of fibrinolytic components and subsequently result in excessive activation of the fibrinolytic cascade (3). In people, this phenomenon has been documented with prostatic adenocarcinoma (4–6). Among individuals with prostatic adenocarcinoma, the prevailing hemostatic disorder is disseminated intravascular coagulopathy (DIC). While prostatic adenocarcinoma-associated hyperfibrinolysis has been documented, it has only been reported to affect up to 1.65% of patients and typically manifests after surgical interventions (3). In veterinary medicine, severe bleeding caused by paraneoplastic disorders with secondary hyperfibrinolysis has not yet been reported. This report demonstrates the development of paraneoplastic secondary hyperfibrinolysis with severe bleeding in a dog with metastatic nasal adenocarcinoma.

## Case description

An 8-year-old male neutered Border Collie (25.7 kg) was serially evaluated at a Veterinary Teaching Hospital (VTH) in January 2023 for recurrent epistaxis.

### Significant medical history

The dog was diagnosed with nasal adenocarcinoma via nasal biopsy, without evidence of metastasis on thoracic or abdominal CT. In June 2022, as part of an ongoing clinical trial, the dog was treated with losartan (10 mg/kg PO q12h) and propranolol (1 mg/kg PO q12h) for 7 days followed by 3 days of stereotactic body radiation therapy (SBRT) using three fractions of 10 Gy photon therapy. Following completion of SRBT, the dog’s treatment regimen was maintained with losartan (10 mg/kg PO q12h), propranolol (1 mg/kg PO q12h), and carprofen (2.2 mg/kg PO q12h). In September 2022 (5 months post-SBRT), repeat CT revealed that the nasal tumor was reduced in size. However, fine-needle aspirates and cytology of the left mandibular lymph node were consistent with metastatic adenocarcinoma. On physical exam, the dog had mild bilateral serosanguinous nasal discharge, which later became more hemorrhagic but self-resolved and was subsequently discharged. Approximately 2 weeks later, the dog was represented to the VTH for re-evaluation, as part of the clinical trial, at which time there was no evidence of nasal discharge. The dog underwent general anesthesia for tumor biopsy after which significant hemorrhage from the bilateral nares was appreciated. Nasal flushing was performed to clear the hemorrhage, and oxymetazoline hydrochloride solution and epinephrine were administered. The dog was subsequently hospitalized with orders for cold compress application and sedation (gabapentin 10 mg/kg PO q12h, trazodone 7 mg/kg PO q8-12h, and acepromazine 0.02 mg/kg PO PRN). The next day, no further epistaxis was appreciated, and the dog was discharged home. No coagulation testing was performed at this visit. In November 2022 (7 months post-SBRT), the dog underwent extirpation of the left mandibular lymph node. Surgery was routine with no adverse events noted in the perioperative or post-operative period. In December 2022 (8 months post-SBRT), a repeat CT scan was performed, and the nasal tumor was decreased further in size. However, enlargement of the retropharyngeal and parotid lymph nodes was noted, and FNA with cytology confirmed further metastatic spread.

### Serial clinical presentations and investigations

In January 2023 (9 months post-SBRT), the dog was presented to the CSU VTH for a reported prolonged unilateral epistaxis for approximately 18 h. Physical examination only confirmed unilateral epistaxis and was otherwise unremarkable. Coagulation testing revealed a markedly prolonged prothrombin time (PT), partial thrombin time (PTT), increased D-dimers, hypofibrinogenemia, mildly increased fibrin degradation product (FDP), and mildly decreased antithrombin (Table 1). On complete blood count (CBC), only a mild leukocytosis [26.9 × 10^3^/μL, reference interval (RI): 4.5–15 × 10^3^/μL] was noted. The biochemical profile showed mild hypoalbuminemia (ALB: 2.9 g/dL, RI: 3–4.3 g/dL) and mild hypomagnesemia (Mg^2+^: 1.7 mg/dL, RI: 1.8–2.4 mg/dL) and was otherwise within normal limits. The dog was hospitalized overnight on aminocaproic acid (ACA) (50 mg/kg IV q6h for four doses), maropitant (1 mg/kg IV q24h), and trazodone (4 mg/kg PO q8h/PRN) and was discharged the next day due to resolution of the epistaxis. The dog was discharged home with ACA (50 mg/kg PO q8h), gabapentin (12 mg/kg PO q8h), and trazodone (4 mg/kg PO q8h/PRN), with instructions to continue administration until the next scheduled recheck visit.

**Table 1:** Serial coagulation profiles in a dog with persistent epistaxis.

	T_1_: 1/20/23	T_5_: 1/24/23	T_7_: 1/26/23	RI	Units
PT	24.8 (H)	73.1 (H)	73.1 (H)	7.4–9.4	s
aPTT	38 (H)	>100 (H)	>100 (H)	9.8–13.3	s
Antithrombin	85 (L)	73 (L)	73 (L)	104–162	%
D-DIMER	1.12 (H)	2.43 (H)	2.43 (H)	0.03–0.4	mcg/mL
Quantitative fibrinogen	<60 (L)	<60 (L)	<60 (L)	123–210	mg/dL
FDP	>5 < 10 (H)	>5 < 10 (H)	>5 < 10 (H)	0–4	mcg/mL

PT, Prothrombin time; aPTT, Activated partial thromboplastin time; FDP, Fibrin degradation product; RI, Reference interval; (H), Above the RI; (L), Below the RI; T_1–7_, Day of treatment with antifibrinolytics.

**Figure 1:**
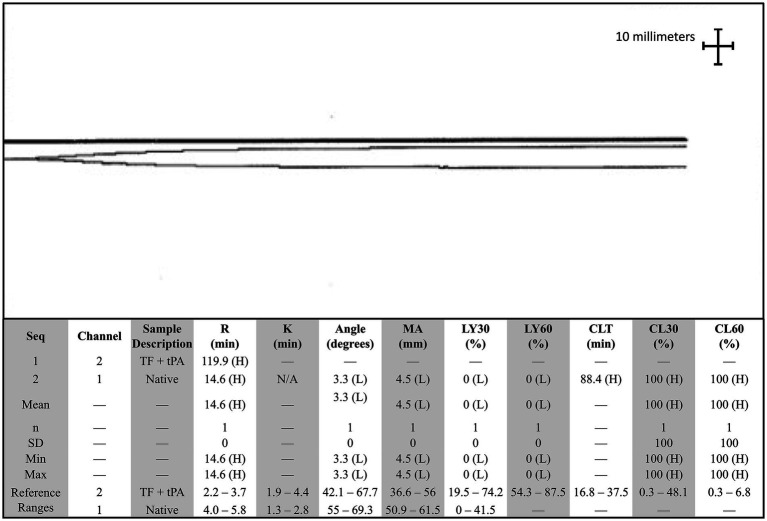
Native and tissue-factor + tissue plasminogen activator thromboelastography (TEG) tracing in a dog with persistent epistaxis. The top tracing is the tissue factor and tissue plasminogen activator TEG tracing, and the bottom tracing is the native tracing. A clot was not able to be formed in the presence of tissue plasminogen activator. Angle, Alpha angle; CLT, Clot lysis time; G, Shear elastic modulus; K, Clot formation time; LY30, Clot Lysis at 30 min; LY60, Clot lysis at 60 min; MA, Maximum amplitude; R, Reaction time; SD, Standard deviation; TEG, Thromboelastography; TF, Tissue factor; tPA, Tissue plasminogen activator.

**Figure 2:**
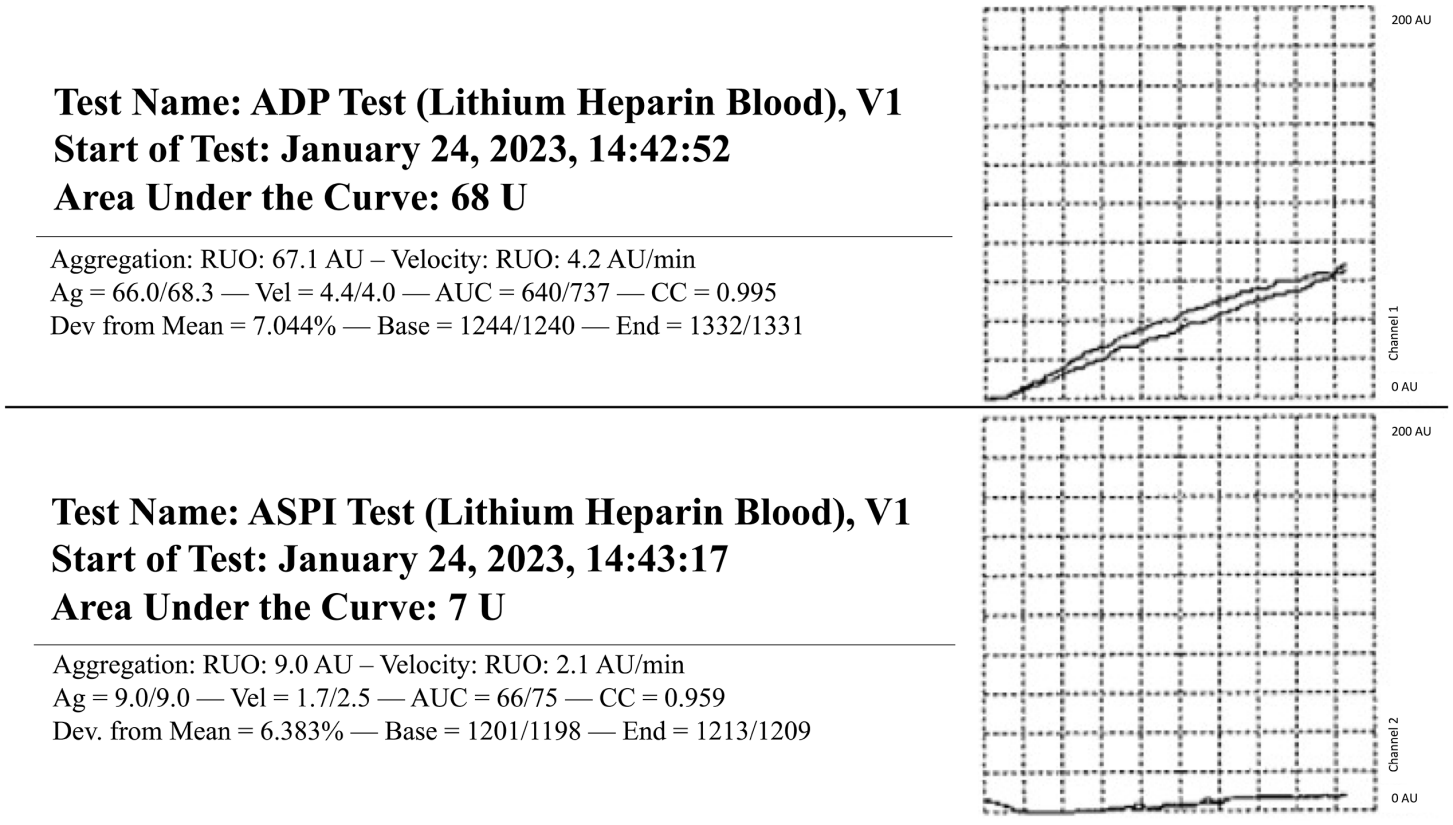
Whole blood impedance platelet aggregometry in a dog with persistent epistaxis. Two platelet agonists were evaluated: adenosine diphosphate (ADP) and arachidonic acid (ASPI). RI, Reference interval. ADP RI (175–294), ASPI RI (131–325).

The dog presented 4 days later for continued bilateral epistaxis. At this visit, laboratory findings revealed that the dog has progressive and strongly regenerative anemia (hematocrit: 26%, RI: 40–55%, hemoglobin: 8 g/dL, RI: 13–20 g/dL, and absolute reticulocyte count: 335.1 × 10^3^/μL, RI: 0–100 × 10^3^/μL), mild thrombocytopenia with platelet clumping noted on blood film review (platelet count: 90 × 10^3^/μL, RI: 200–500 × 10^3^/μL), static leukocytosis (WBC: 27.7 × 10^3^/μL, RI: 4.5–15 × 10^3^/μL), mild hypoglycemia (glucose: 64 mg/dL, RI: 70–115 mg/dL), and mild hypoalbuminemia (albumin: 2.8 g/dL, RI: 3–4.3 g/dL). Thoracic radiographs revealed a newly identified pulmonary nodule. A coagulation panel revealed a progressively worsened coagulopathy with continued hypofibrinogenemia (Table 1). Native thromboelastography (TEG) (using no activators) demonstrated a prolonged initiation to clot formation, in addition to delayed clot kinetics and an extremely weak clot (Figure 1). A clot was not able to be formed in the tissue-factor + tPA TEG tracing (Figure 1). Platelet aggregometry was performed with a multiple electrode aggregometer (Multiplate 5.0 Analyzer, DiaPharma Group Inc., West Chester, Ohio) using whole blood impedance aggregometry and showed decreased aggregation compared with institution reference ranges in the presence of two platelet agonists [adenosine diphosphate (ADP) and arachidonic acid (ASPI)] (Figure 2). The dog was hospitalized on fluid therapy, maropitant (1 mg/kg IV q24h), and ACA (50 mg/kg IV q6h) as initial treatments. However, given aforementioned findings, the dog received a fresh frozen plasma (FFP) transfusion (250 mL IV over 4 h, 10 mL/kg), and vitamin K_1_ (5 mg/kg SC q12h) was added to the treatments. On the second day of hospitalization, repeat testing found progressive anemia [packed cell volume (PCV): 19%, RI: 29–51%], and the dog developed hyperkinetic femoral pulses, decreased mentation, and progressive lethargy. An abdominal ultrasound was performed which showcased a scant amount of anechoic peritoneal effusion, not a sufficient quantity for sampling. Although the gallbladder exhibited a small volume of echogenic debris in its lumen, the rest of the abdomen, including the spleen and liver, appeared ultrasonographically normal. Given the clinical anemia progression and evidence of hypovolemia, packed red blood cell transfusion (10 mL/kg IV for 4 h), additional FFP transfusion (10 mL/kg IV for 4 h), and two units of cryoprecipitate (1.2 mL/kg IV for 1 h) were administered. After those treatments, the dog demonstrated marked improvement in circulatory parameters, non-appreciable epistaxis, and improved anemia (PCV: 29%, RI: 29–51%). On the third day of hospitalization, the dog’s recheck coagulation profile remained unchanged (Table 1). Given the clinical improvements, the owner elected to take the dog home at that time, and the dog was discharged from the VTH with instructions to transition to oral vitamin K_1_ and ACA at the aforementioned doses.

The next day following discharge, the dog was represented for severe lethargy, bilateral epistaxis, and new serosanguinous oral discharge. Given the poor prognosis and perceived decline in quality of life, the owner elected for humane euthanasia. Before euthanasia, additional blood was sampled for specific coagulation factor, fibrinogen, and von Willebrand factor analysis which revealed normal factor II and von Willebrand factor, decreased factors VII, VIII, and X, and a severely decreased fibrinogen (Table 2). Necropsy was performed which found metastatic nasal adenocarcinoma in the nasal sinuses, regional lymph nodes, and lungs. The nasal mass, located within the nasal sinus, was decalcified and found to contain neoplastic epithelial cells, accompanied by significant osteonecrosis characterized by irregular bony trabeculae lacking osteocytes within lacunae, as well as scalloped trabeculae displaying deeply basophilic reversal lines. Additionally, the nasal turbinates showed an invasive, densely cellular mass of neoplastic epithelial cells, featuring solid and occasionally tubular formations, moderate anisocytosis and anisokaryosis, high mitotic activity (>100 per 10 high-power fields), multifocal necrosis with associated inflammatory infiltrate, and scattered regions of well-differentiated respiratory epithelium and submucosa. Examination of the right retropharyngeal lymph node revealed that nodal architecture is expanded by a multilobulated mass comprised of neoplastic epithelial cells, which are similar to those of the nasal mass and turbinates. In the lung, focally, the pulmonary parenchyma is expanded by a nodule comprised of neoplastic epithelial cells. There are multifocal tumor emboli within the lymphovascular structures and pulmonary vessels. In the liver, chronic moderate midzonal to periportal hepatocellular degeneration with a focal intravascular tumor embolus was identified.

**Table 2:** Specific factor activity levels, fibrinogen, and vWF analysis in a dog with persistent epistaxis.

Variable	Result	RI	Units
Factor II Coagulant Activity (FII:C)	57	50–150	%
Factor VII Coagulant Activity (FVII:C)	36 (L)	50–150	%
Factor VIII Coagulant Activity (FVIII:C)	40 (L)	50–200	%
Factor X Coagulant Activity (FX:C)	61 (L)	80–175	%
Fibrinogen	<11 (L)	150–490	mg/dL
von Willebrand Factor (vWF:Ag)	85	70–180	%

Analyses were performed, and reference ranges were also provided by the Cornell Comparative Coagulation Laboratory. vWF, von Willebrand Factor; Ag, Antigen; (H), Above the RI; (L), Below the RI; RI, Reference interval.

## Discussion

Disseminated intravascular coagulopathy is a complex disorder characterized by abnormal hemostatic activation, with clinical manifestation depending on the cause. Laboratory diagnosis of DIC is characterized by decreased platelet count, prolonged PT, prolonged aPTT, decreased plasma fibrinogen concentration, decreased plasma antithrombin (AT) activity, increased D-dimers, and increased plasma FDP (7, 8). While these criteria help identify DIC in veterinary medicine, this description does not encompass the various DIC phenotypes observed in human medicine. The International Society on Thrombosis and Hemostasis (ISTH) characterizes two distinct phenotypes of DIC: thrombotic and fibrinolytic. The thrombotic form often leads to organ dysfunction due to clotting, while the fibrinolytic form causes severe bleeding due to excessive clot breakdown (9, 10). In many cases, hemostatic abnormalities can precede clinical and diagnostic evidence of DIC and are reportedly triggered by a multitude of causes, including cancer (11–13). In veterinary medicine, such severe bleeding secondary to hyperfibrinolysis has not been reported in association with disseminated malignancy.

Primary sinonasal neoplasms are relatively uncommon, accounting for less than 2% of all known canine cancers (14, 15). The typical clinical signs include epistaxis, facial deformities, regional lymphadenopathy, and nasal airflow obstruction (16). Fortunately, these clinical signs often respond well with appropriate therapy. Interestingly, Maruyama et al. (7) have observed an association between DIC and dogs with solid malignant tumors, occurring in 9.6% of cases (20/208). Among these cases, 18 dogs were diagnosed with nasal adenocarcinoma, and the majority (16 of 18) showed no clinical or clinicopathologic evidence of DIC (7). This data suggest that delayed, spontaneous severe bleeding in dogs with nasal adenocarcinoma is uncommon.

When considering the present case report, approximately 5 months after radiotherapy, the first bleeding event was noted after tumor biopsy. At that time, no coagulation or platelet function testing was performed, and the excessive bleeding ceased overnight without any additional interventions. When the dog was represented 9 months after radiotherapy for persistent epistaxis, coagulation testing was suggestive of DIC and hyperfibrinolysis (Table 1). In human medicine, malignancy-associated DIC is categorized as subacute, acute, and chronic and has been most often associated with prostatic adenocarcinomas (4–6, 17–19). While the mechanisms underlying the association between cancer and DIC are multifactorial and partially understood, the most prominent theory centers on uPA acting as a primary agent in malignancy-associated DIC (19). uPA has been associated with cancer cell migration, invasion, and modulation of cell adhesion, thereby promoting the formation and progression of various cancers (19, 20). uPA is also known to catalyze the conversion of plasminogen into plasmin without negative feedback, resulting in the breakdown of fibrin clots and the degradation of the extracellular matrix (18, 21, 22). This pathogenesis results in the development of secondary hyperfibrinolysis as the resultant hypofibrinogenemia is the result of imbalanced excessive activation of structurally normal fibrinolytic enzymes or enhanced susceptibility of fibrin to proteolysis (23). In this case, the dog also had deficiencies in factors VII, VIII, and X, along with severe hypofibrinogenemia, all of which are suggestive of an impaired ability to form a clot and hemostatic dysfunction (Table 2). Moreover, the reduction in factor VIII specifically has been associated with upregulation of the fibrinolytic system, resulting in secondary hyperfibrinolysis (23). Thus, when taken in tandem with the dog’s history of confirmed metastatic adenocarcinoma, thrombocytopenia, regenerative anemia, hypocoagulable profile on TEG (Figure 1), and decreased platelet aggregation on impedance aggregometry (Figure 2), the most likely cause for the dog’s progressive clinical decline is chronic DIC with a concurrent paraneoplastic secondary hyperfibrinolysis, a rare diagnosis in veterinary medicine (21).

When exploring other reasons for severe bleeding, severe hypofibrinogenemia is unusual and rarely linked to neoplasia in veterinary medicine or more specifically with dogs with nasal adenocarcinoma. Differentials such as primary hyperfibrinolysis, acquired hypofibrinogenemia, and late-radiation toxicity were considered as potential causes for the dog’s excessive bleeding. In this case, the dog had severe hypofibrinogenemia, which could represent primary hyperfibrinolysis, or an absolute quantitative or qualitative abnormality of proteins was directly involved in the fibrinolytic process (23). However, primary hyperfibrinolysis typically occurs with increased FDP values and normal D-dimers, which would not fit with coagulation abnormalities of the dog. Moreover, TEG analysis which is indicative of primary hyperfibrinolysis would showcase rapid clot formation and subsequent rapid clot lysis (24). In this case, this dog’s TEG profile revealed a prolonged initiation to clot formation, delayed clot kinetics, and a weak clot, consistent with fibrin degradation and DIC or secondary hyperfibrinolysis, further supporting the notion of a secondary hyperfibrinolysis, as observed in the context of underlying malignancy-induced coagulopathy. Next, due to cytotoxic agents (chemotherapy), hypofibrinogenemia is considered as it has been linked to acute myeloid leukemia in people (25). Notably, this phenomenon has never been reported in veterinary medicine. Moreover, in this case, the dog had not received any cytotoxic chemotherapeutic agents. Instead, immunotherapy and radiation therapy had been pursued for approximately 9 months prior to the bleeding events, and thus, it was also excluded as a differential. Other causes for this type of severe hypofibrinogenemia and other coagulation abnormalities could have included synthetic liver failure, but clinically, diagnostically, or histologically there was no evidence to support this diagnosis. Finally, we also considered late radiation toxicity after radiotherapy, but it was deemed unlikely due to the delayed onset of bleeding, unusual tissue response, and the presence of other clotting abnormalities. Irrespective of the etiology, the treatment of choice for hypofibrinogenemia is cryoprecipitate, which contains factor VIII, von Willebrand factor (vWF), fibrinogen, factor XIII, and fibronectin (26). In this case, treatments such as cryoprecipitate infusions, multiple FFP transfusions, and several days of ACA administration were short lived, further reinforcing the diagnosis of severe secondary paraneoplastic hyperfibrinolysis (27–29).

Finally, regenerative anemia and mild thrombocytopenia of the dog could have represented a response to ongoing hemorrhage experienced for multiple days. The decreased platelet aggregability that was noted in this patient is suspected to be related to several factors such as a consumptive process from the severe epistaxis, an *in vitro* change due to the thrombocytopenia, and platelet dysfunction secondary to metastatic neoplasia (30–33).

## Conclusion

This case report presents the first documented instance of paraneoplastic secondary hyperfibrinolysis in a dog with metastatic nasal adenocarcinoma, necessitating multiple blood product transfusions and a prolonged course of ACA due to severe and recurrent bleeding. Although coagulopathies account for less than 5% of cases in dogs with epistaxis (34), the literature suggests that coagulopathies occur in approximately 56% of dogs with malignant neoplasia (7). As such, clinicians should consider systematic coagulation testing in patients with malignant neoplasia presenting with acute bleeding episodes. Such coagulation profiles can guide the administration of anti-fibrinolytic medications and blood products.

## Data availability statement

The original contributions presented in the study are included in the article/supplementary material, further inquiries can be directed to the corresponding author.

## Ethics statement

Ethical approval was not required for the studies involving animals in accordance with the local legislation and institutional requirements because this animal study is a case report. Written informed consent was obtained from the owners for the participation of their animals in this study.

## Author contributions

KG: Conceptualization, Data curation, Formal analysis, Investigation, Methodology, Project administration, Resources, Writing – original draft, Writing – review & editing. TP: Conceptualization, Data curation, Formal analysis, Investigation, Methodology, Project administration, Resources, Writing – original draft, Writing – review & editing. M-KB: Conceptualization, Data curation, Investigation, Methodology, Software, Supervision, Writing – review & editing. LG: Writing – review & editing. SS: Conceptualization, Data curation, Formal analysis, Investigation, Methodology, Resources, Software, Supervision, Writing – review & editing.

## Glossary

ACA: Aminocaproic Acid
ADP: Adenosine diphosphate
ALB: Albumin
aPTT: Activated partial thromboplastin time
ASPI: Arachidonic acid
AT: Antithrombin
CT: Computed tomography
DD: D-Dimers
DIC: Disseminated intravascular coagulopathy
FDP: Fibrin degradation product
FFP: Fresh frozen plasma
Gy: Gray (unit of radiation dose)
ISTH: International Society on Thrombosis and Hemostasis
PCV: Packed cell volume
PT: Prothrombin time
RI: Reference interval
SBRT: Stereotactic body radiation therapy
TEG: Thromboelastography
tPA: Tissue-type plasminogen activator
uPA: Urokinase-type plasminogen activator
vWF: Von Willebrand factor

## References

[1] Iba T Levy JH Thachil J Wada H Levi M. The progression from coagulopathy to disseminated intravascular coagulation in representative underlying diseases. Thromb Res. (2019) 179:11–4. doi: 10.1016/j.thromres.2019.04.030, PMID: 31059996

[2] Camerer E Qazi AA Duong DN Cornelissen I Advincula R Coughlin SR. Platelets, protease-activated receptors, and fibrinogen in hematogenous metastasis. Blood. (2004) 104:397–401. doi: 10.1182/blood-2004-02-0434, PMID: 15031212

[3] Okajima K Kohno I Soe G Okabe H Takatsuki K Binder BR. Direct evidence for systemic fibrinogenolysis in patients with acquired α2-plasmin inhibitor deficiency. Am J Hematol. (1994) 45:16–24. doi: 10.1002/ajh.2830450104, PMID: 8250008

[4] Kulić A Cvetković Z Libek V. Primary hyperfibrinolysis as the presenting sign of prostate cancer: a case report. Vojnosanit Pregl. (2016) 73:877–80. doi: 10.2298/VSP150525076K, PMID: 29320623

[5] Jafri MA Cohen JV Much MA Petrylak DP Podoltsev NA. A patient with pancytopenia, intractable epistaxis, and metastatic prostate cancer: how correct diagnosis of primary hyperfibrinolysis helps to stop the bleeding. Clin Genitourin Cancer. (2016) 14:e545–8. doi: 10.1016/j.clgc.2016.05.002, PMID: 27320762

[6] Hyman DM Soff GA Kampel LJ. Disseminated intravascular coagulation with excessive fibrinolysis in prostate cancer: a case series and review of the literature. Oncology. (2011) 81:119–25. doi: 10.1159/000331705, PMID: 21986538

[7] Maruyama H Miura T Sakai M Koie H Yamaya Y Shibuya H. The incidence of disseminated intravascular coagulation in dogs with malignant tumor. J Vet Med Sci. (2004) 66:573–5. doi: 10.1292/jvms.66.573, PMID: 15187373

[8] Stokol T. Plasma D-dimer for the diagnosis of thromboembolic disorders in dogs. Vet Clin Small Anim Pract. (2003) 33:1419–35. doi: 10.1016/S0195-5616(03)00096-2, PMID: 14664206

[9] Wada T Gando S. Phenotypes of disseminated intravascular coagulation. Thromb Haemost. (2023) 100:958–9. PMID: 37657485 10.1055/a-2165-1142 PMC10890912

[10] Levi M. Disseminated intravascular coagulation in cancer: an update In: Seminars in Thrombosis and Hemostasis. New York, NY, USA: Thieme Medical Publishers (2019). 10.1055/s-0039-1687890 31041800

[11] Falanga A Panova-Noeva M Russo L. Procoagulant mechanisms in tumour cells. Best Pract Res Clin Haematol. (2009) 22:49–60. doi: 10.1016/j.beha.2008.12.009 19285272

[12] Lu G Jia L Yang R Lv Z Cui J. Acquired hyperfibrinolysis as the presenting sign of metastatic breast cancer: a case report. Oncol Lett. (2023) 25:1–6. doi: 10.3892/ol.2023.13692 36817045 PMC9933151

[13] Winther-Larsen A Sandfeld-Paulsen B Hvas A-M. Hyperfibrinolysis in patients with solid malignant neoplasms: a systematic review In: Seminars in Thrombosis and Hemostasis, New York, NY 10001, USA: Thieme Medical Publishers, Inc. (2021). vol. 47:581–8. 32968992 10.1055/s-0040-1715795

[14] Madewell B Priester W Gillette E Snyder S. Neoplasms of the nasal passages and paranasal sinuses in domesticated animals as reported by 13 veterinary colleges. Am J Vet Res. (1976) 37:851–6. PMID: 937809

[15] Mortier J Blackwood L. Treatment of nasal tumours in dogs: a review. J Small Anim Pract. (2020) 61:404–15. doi: 10.1111/jsap.13173, PMID: 32715503

[16] Strasser JL Hawkins EC. Clinical features of epistaxis in dogs: a retrospective study of 35 cases (1999–2002). J Am Anim Hosp Assoc. (2005) 41:179–84. doi: 10.5326/0410179, PMID: 15870252

[17] Smith JA Jr Soloway MS Young MJ. Complications of advanced prostate cancer. Urology. (1999) 54:8–14. doi: 10.1016/S0090-4295(99)00448-3 10606278

[18] Prokopchuk-Gauk O Brose K. Tranexamic acid to treat life-threatening hemorrhage in prostate cancer associated disseminated intravascular coagulation with excessive fibrinolysis. Cureus. (2015) 7:e428. doi: 10.7759/cureus.428 26848417 PMC4727914

[19] Ong SY Taverna J Jokerst C Enzler T Hammode E Rogowitz E. Prostate cancer-associated disseminated intravascular coagulation with excessive fibrinolysis treated with degarelix. Case Rep Oncol Med. (2015) 2015:1–6. doi: 10.1155/2015/212543 PMC4646996 26613055

[20] Mahmood N Mihalcioiu C Rabbani SA. Multifaceted role of the urokinase-type plasminogen activator (uPA) and its receptor (uPAR): diagnostic, prognostic, and therapeutic applications. Front Oncol. (2018) 8:24. doi: 10.3389/fonc.2018.00024, PMID: 29484286 PMC5816037

[21] Zoia A Drigo M Caldin M Simioni P Piek CJ. Fibrinolysis in dogs with Intracavitary effusion: a review. Animals. (2022) 12:2487. doi: 10.3390/ani12192487, PMID: 36230236 PMC9558497

[22] Chapin JC Hajjar KA. Fibrinolysis and the control of blood coagulation. Blood Rev. (2015) 29:17–24. doi: 10.1016/j.blre.2014.09.003, PMID: 25294122 PMC4314363

[23] Kolev K Longstaff C. Bleeding related to disturbed fibrinolysis. Br J Haematol. (2016) 175:12–23. doi: 10.1111/bjh.14255, PMID: 27477022 PMC5096260

[24] Carey M Cressey DM. Hyperfibrinolysis–is it common? Measurement and treatment including the role of thromboelastography. Clin Risk. (2009) 15:188–91. doi: 10.1258/cr.2009.090028

[25] Elamil ZG Tuncer HH Tara R Homsi SAA. Consistent Hypofibrinogenemia associated with induction anti-tumor chemotherapy for acute myeloid leukemia. Am Soc Hematol. (2010) 116:1400. doi: 10.1182/blood.V116.21.1400.1400

[26] Pantanowitz L Kruskall MS Uhl L. Cryoprecipitate: patterns of use. Am J Clin Pathol. (2003) 119:874–81. doi: 10.1309/56MQVQAQG8YU90X9 12817436

[27] Plaut D. The laboratory's role in disseminated intravascular coagulation. J Contin Educ Top Issues. (2011) 13:24–9.

[28] Fisher VR Scott MK Tremblay CA Beaulieu GP Ward DC Byrne KM. Disseminated intravascular coagulation: laboratory support for management and treatment. Lab Med. (2013) 44:e10–4. doi: 10.1309/LMTCAVNHBUGWTP7K

[29] Toh C-H Alhamdi Y Abrams ST. Current pathological and laboratory considerations in the diagnosis of disseminated intravascular coagulation. Ann Lab Med. (2016) 36:505–12. doi: 10.3343/alm.2016.36.6.505, PMID: 27578502 PMC5011102

[30] Crispin P Gardiner EE. Platelets and cancer… the plot doesn’t always thicken. J Thromb Haemost. (2020) 18:2482–5. doi: 10.1111/jth.14944 33460258

[31] Braun A Anders H-J Gudermann T Mammadova-Bach E. Platelet-cancer interplay: molecular mechanisms and new therapeutic avenues. Front Oncol. (2021) 11:665534. doi: 10.3389/fonc.2021.665534, PMID: 34322381 PMC8311658

[32] Sweeney JD Labuzetta JW Fitzpatrick JE. The effect of the platelet count on the aggregation response and adenosine triphosphate release in an impedance lumi-aggregometer. Am J Clin Pathol. (1988) 89:655–9. doi: 10.1093/ajcp/89.5.655, PMID: 3358370

[33] Müller MR Salat A Pulaki S Stangl P Ergun E Schreiner W. Influence of hematocrit and platelet count on impedance and reactivity of whole blood for electrical aggregometry. J Pharmacol Toxicol Methods. (1995) 34:17–22. doi: 10.1016/1056-8719(94)00075-F, PMID: 7496042

[34] Bissett SA Drobatz KJ McKnight A Degernes LA. Prevalence, clinical features, and causes of epistaxis in dogs: 176 cases (1996–2001). J Am Vet Med Assoc. (2007) 231:1843–50. doi: 10.2460/javma.231.12.1843, PMID: 18081523

